# Immunohistochemical Evaluation of Histological Change in a Chinese Milroy Disease Family With Venous and Skin Abnormities

**DOI:** 10.3389/fgene.2019.00206

**Published:** 2019-03-19

**Authors:** Sijia Zhang, Xihui Chen, Lijuan Yuan, Shuyan Wang, Dangzhi Moli, Shujuan Liu, Yuanming Wu

**Affiliations:** ^1^Department of Biochemistry and Molecular Biology, Center for DNA Typing, Air Force Medical University, Xi'an, China; ^2^State Key Laboratory of Military Stomatology and National Clinical Research Center for Oral Diseases and Shaanxi Engineering Research Center for Dental Materials and Advanced Manufacture, Department of Implant Dentistry, School of Stomatology, Air Force Medical University, Xi'an, China; ^3^Department of General Surgery, Tangdu Hospital, Air Force Medical University, Xi'an, China; ^4^Department of Obstetrics and Gynecology, Xijing Hospital, Air Force Medical University, Xi'an, China

**Keywords:** Milroy disease, lymphedema, D2-40, fetus, FLT4

## Abstract

**Background:** Milroy disease (MD) is rare and autosomal dominant resulting from mutations of the vascular endothelial growth factor receptor-3 *(VEGFR-3 or FLT4)*, which leads to dysgenesis of the lymphatic system.

**Methods:** Here we report a Chinese MD family with 2 affected members of two generations. We identified the mutation of c.3075G>A in one allele of FLT4 in Chinese population firstly. The father and child presented lymphedema under knees both. Unfortunately, the child was premature delivered for a car accident of the mother and then died of asphyxia. Then we gathered the tissue of the lower-limb from the child with permission from the parents and ethic committee. We stained the tissue with lymphatic marker D2-40 and hematoxylin-eosin to explore the histological changes. Afterwards, we compared the results with a normal child who unfortunately died of premature delivery also.

**Results:** It is firstly identified the mutation of FLT4: c.3075G>A in Chinese population, and the mutation Inherited in the lineage. The histological evaluation indicated: (1) The number of lymphatic vessels decreased; (2) The morphology and structure of lymphatic vessels was abnormal. And what is added to our knowledge: (1) Capillary hyperemia and phlebectasia is severe; (2) Vascular malformations; (3) The number of vascular endothelial cells and vascular smooth muscle cells decreased; (4) Large sheets of epidermis desquamated; (5) The numbers of cutaneous appendages reduced in MD.

**Conclusions:** Based on the new findings, we assume that mutation of FLT4 not only affect the lymphogenesis, but also the angiogenesis, and epidermis structure.

## Background

Milroy disease *(MD; hereditary lymphedema type I; MIM# 153100)* is first described by Milroy in 1892. It is known caused by the dysfunction of the lymphatic system so far, with the key features of congenital onset and primary lymphedema (Milroy, 1892). The estimated incidence is 1/6,000 worldwide, with a male/female ratio of 1:2.3 generally (Gezginc et al., 2012). Typical MD patients usually exhibit lymphedema at birth with swelling of the lower-limb, most times it is bilateral. The patients often have a brawny texture of the skin and hyperkeratosis is reported from the laboratory. The swelling is confined to the dorsum of the foot with deep skin creases which could be detected on the toes.

The gene locus of MD was first reported by Ferrell and Evans at chromosomal location 5q35 (Ferrell et al., 1998; Evans et al., 1999). The mutated gene *(FLT4)* in this region encodes a tyrosine kinase receptor for vascular endothelial growth factors C *(VEGFR-3)*. The protein is believed to be involved in the process of lymphangiogenesis and maintenance of the lymphatic endothelium (Brice et al., 2005). Mutations of *FLT4* is responsible for about 75% of the diagnosed MD cases which has been published (Connell et al., 2009). Recent studies showed that the patients had large caliber great saphenous veins while presented no cutaneous signs of venous disease (Gordon et al., 2013). However, the superficial venous valve reflux indicates the venous development of MD patients might also be abnormal (Mellor et al., 2010).

For years, the limitations in the methods restrict us to present lymphatic vessels in MD patients, and the pathogenesis was mainly based on the evidence of lymphatic imaging, animal and cell studies. It is reported that the D2-40 monoclonal antibody could selectively detect lymphatic vessels (Bai et al., 2013), for it can specifically combine to a fixation-resistant epitope on a 40 kDa O-linked sialoglycoprotein which is expressed in lymphatic endothelium but not in blood vessels (Yonemura et al., 2006). So, it could help us present the histological change in MD patients clearly.

Following, we will report one Chinese MD family with typical symptoms. Helping by the next-generation sequencing *(NGS)*, immune-histo-chemical staining with D2-40 and hematoxylin-eosin *(HE)*, we represent histological change of the lower-limb and the feet dorsum from the dead child patient *(premature death, 28 weeks* + *2 days)* in this family, with the mutation in the FLT4 *(c.3075G*>*A, exon 22)*. Then, we compared the results with a normal child who unfortunately died of premature delivery also. The results offered us a new perspective to understand the occurrence and development of lymphedema in MD patients.

## Case Presentation

All the operations and tests were given full authorization by the members from two families. And the study was full authorized by the ethic committee of the Air force medical university. All the operations were under supervision by one member of the families and one officer of the ethic committee. Written and informed consent was obtained from the guardians of the patient for publication of this case report.

### Clinical Characterization of the Family (Figure 1)

#### Child Patient

Male, died of neonatal asphyxia post-delivery. The B-ultrasound scanner showed bilateral lower limb edema in regular pregnancy test, which was confirmed post-delivery. The skin color of the lower limbs was slightly purple (Figure 2a). The father *(proband)* developed edema in both lower limbs from birth, and had no other physical dysfunction. The mother denied any history of illness or medication during pregnancy. The grandparents *(paternal)* also had no physical dysfunction.

**Figure 1:**
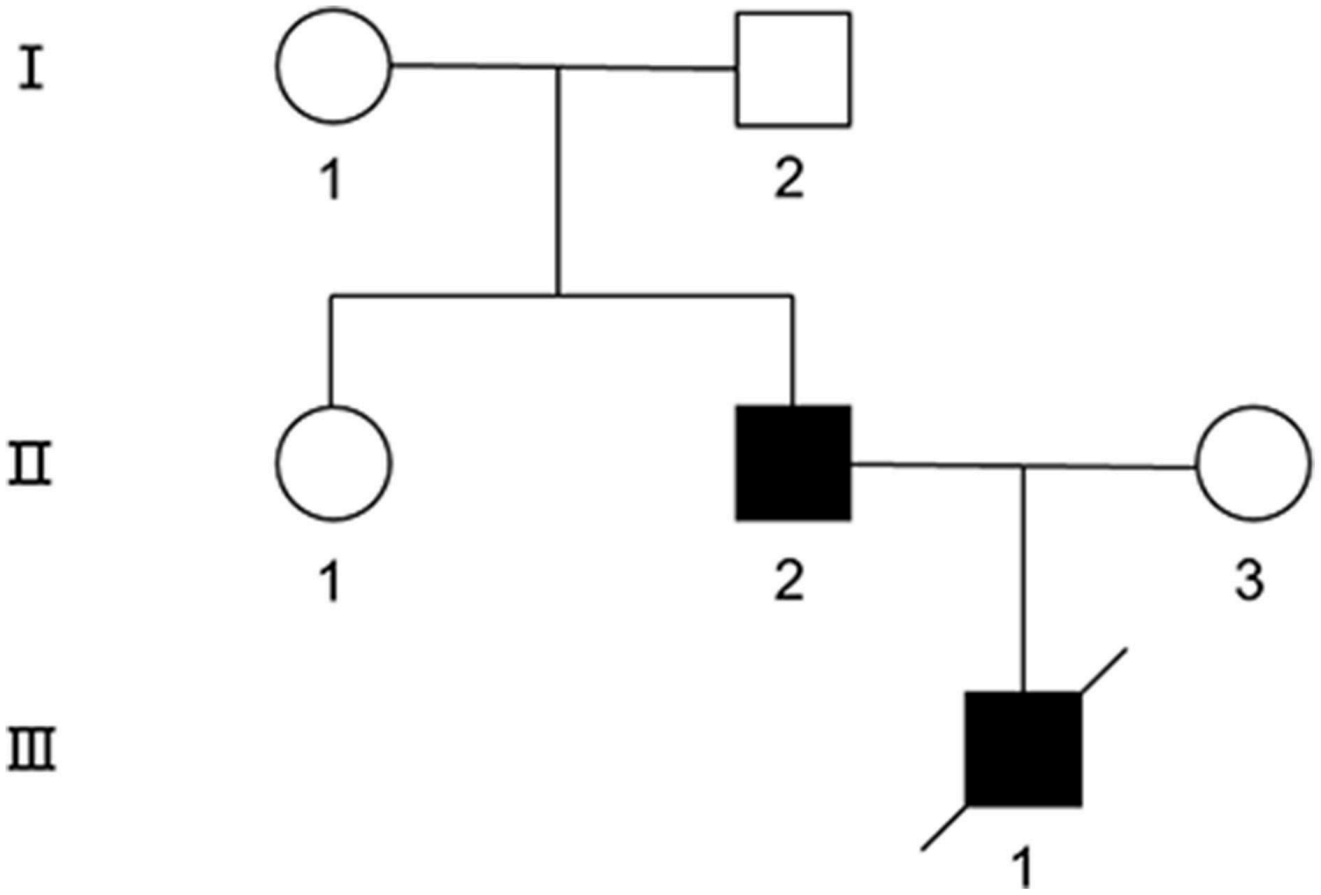
The pedigree of the MD family. Symbols are as follows: square, male; circle, female; filled, affected; empty, unaffected.

**Figure 2:**
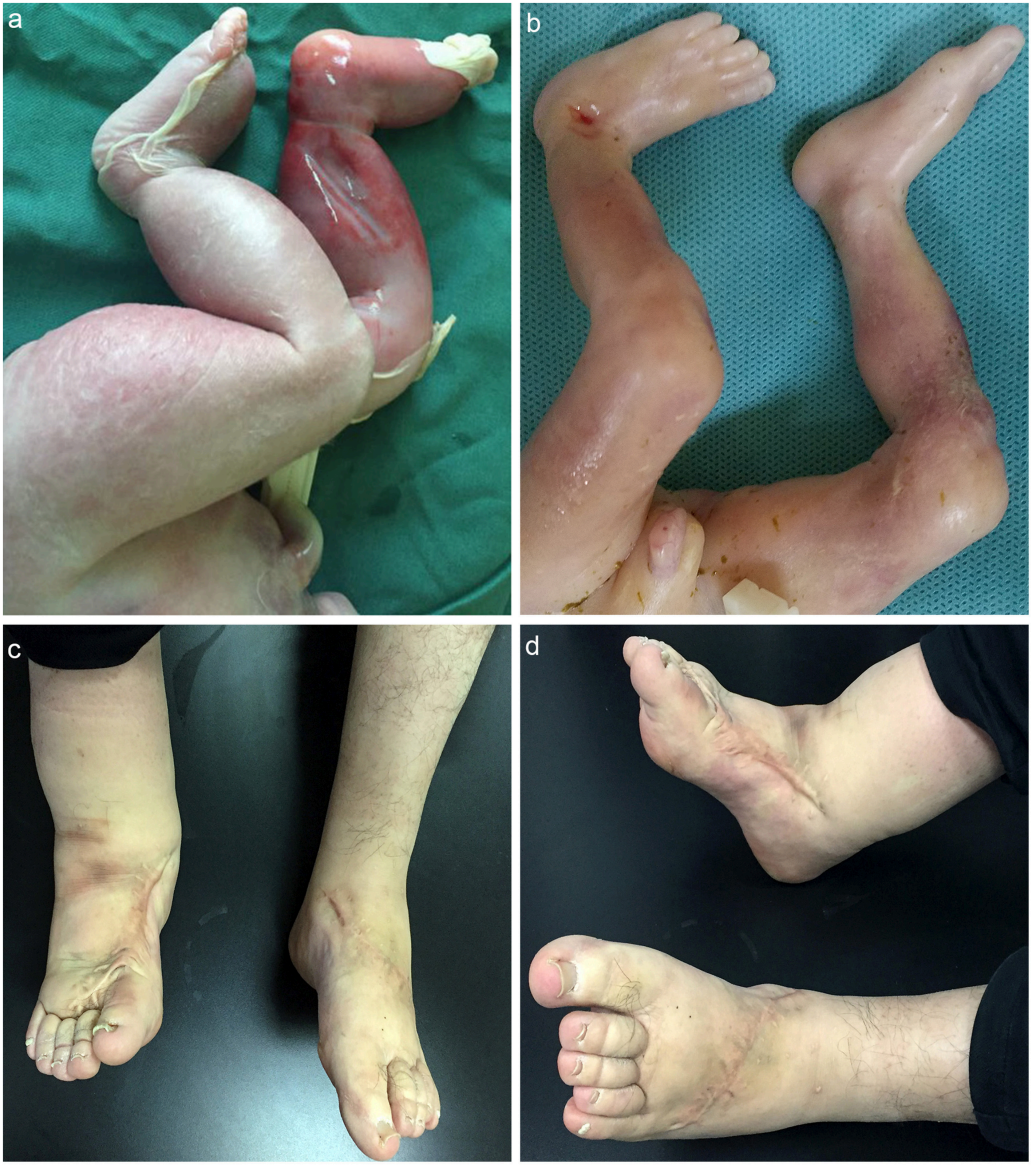
**(a)** MD child patient, the skin color of the lower limbs was slightly purple, edema in both lower limbs could be detected; **(b)** matched child, no obvious abnormality; **(c,d)** father patient, edema in right lower limb could be detected; the left lower limb became normal post plastic surgery.

#### Mother

Gestational age 28 weeks + 2 days, premature delivery for car accident, number and structure of Chromosome showed nothing abnormal post amniocentesis.

#### Father (Proband)

The skin temperature was normal, there was no limitation of movement, and the muscle tension of the limbs was normal. There was no obvious abnormality in blood gas, blood biochemistry, blood routine, thyroid function, blood lead, urine routine, urine, and blood metabolism. Microvirus B19 DNA and nucleic acid test resulted negative. Quantitative detection of CMV- PP65 antigen and CMV- DNA was also negative.

The father was diagnosed as MD since birth and received plastic surgery, the symptoms of the left lower-limb was reduced while no significant improvement showed in the right. The father was also advised to order stretch socks to relieve symptoms (Figures 2c,d) (Supplementary Video 1).

#### Other Family Members (Paternal)

Aunt and grandparents showed nothing abnormal.

### Clinical Characterization of the Matched Child

#### Mother

The mother was diagnosed of gastric carcinoma 25 weeks since menelipsis, and the family asked for termination of pregnancy to save the mother. The gestational age was 26 weeks + 5 days, and the number and structure of Chromosome showed nothing abnormal post amniocentesis.

#### Matched Child

Male, died of labor induction. The structure and skin color of the lower limbs was normal, and no other physical dysfunction was detected (Figure 2b).

## Description of Laboratory Investigations and Diagnostic Tests

### D2-40 Staining and HE Staining

The tissue was gathered from the swollen situs of the lower-limb and the feet dorsum. Then we preserved them in 10% formalin, paraffin-embedded after dehydration. Afterwards, we used the standard staining method to stain the tissue slice. We examined the sections under stereomicroscope *(DMI6000 B, Leica Microsystems, Shanghai, China)*.

#### Results of D2-40 Staining (Figure 3)

1. The number of lymphatic vessels decreased in Milroy disease child.
2. The abnormal morphology and structure of lymphatic vessels could be detected in MD child.

**Figure 3:**
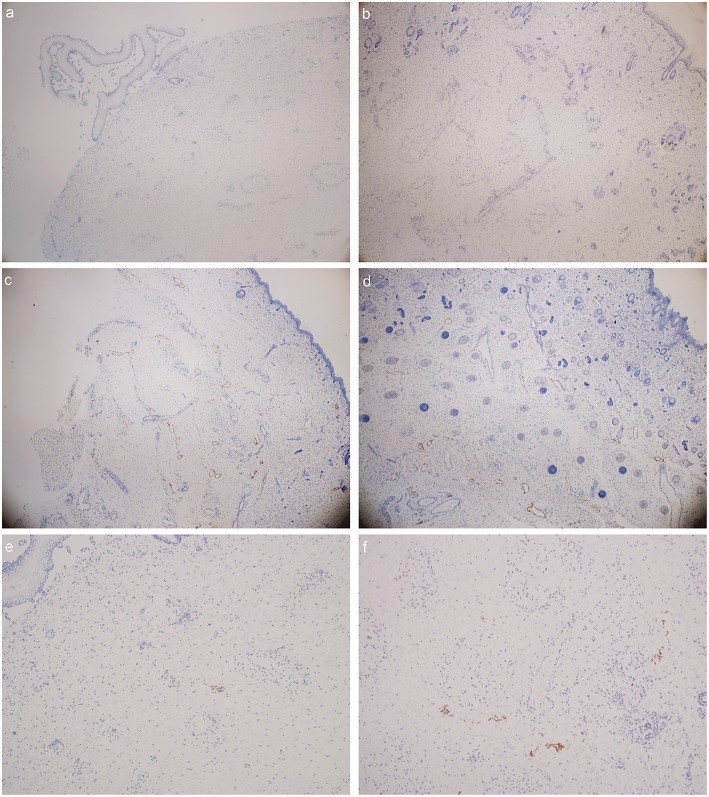
D2-40 stain (lymphatic vessels were presented in orange color): **(a)** foot dorsum of MD child (40 times magnification); **(b)** lateral low-limb skin of MD child (40 times magnification); **(c)** foot dorsum of matched child (40 times magnification); **(d)** lateral low-limb skin of matched child (40 times magnification). **(e,f)** lymphatic vessels found within the field of vision **(e)** foot dorsum of MD child (100 times magnification); **(f)** lateral low-limb skin of MD child (100 times magnification).

#### Results of HE Staining

**Figure 4:**
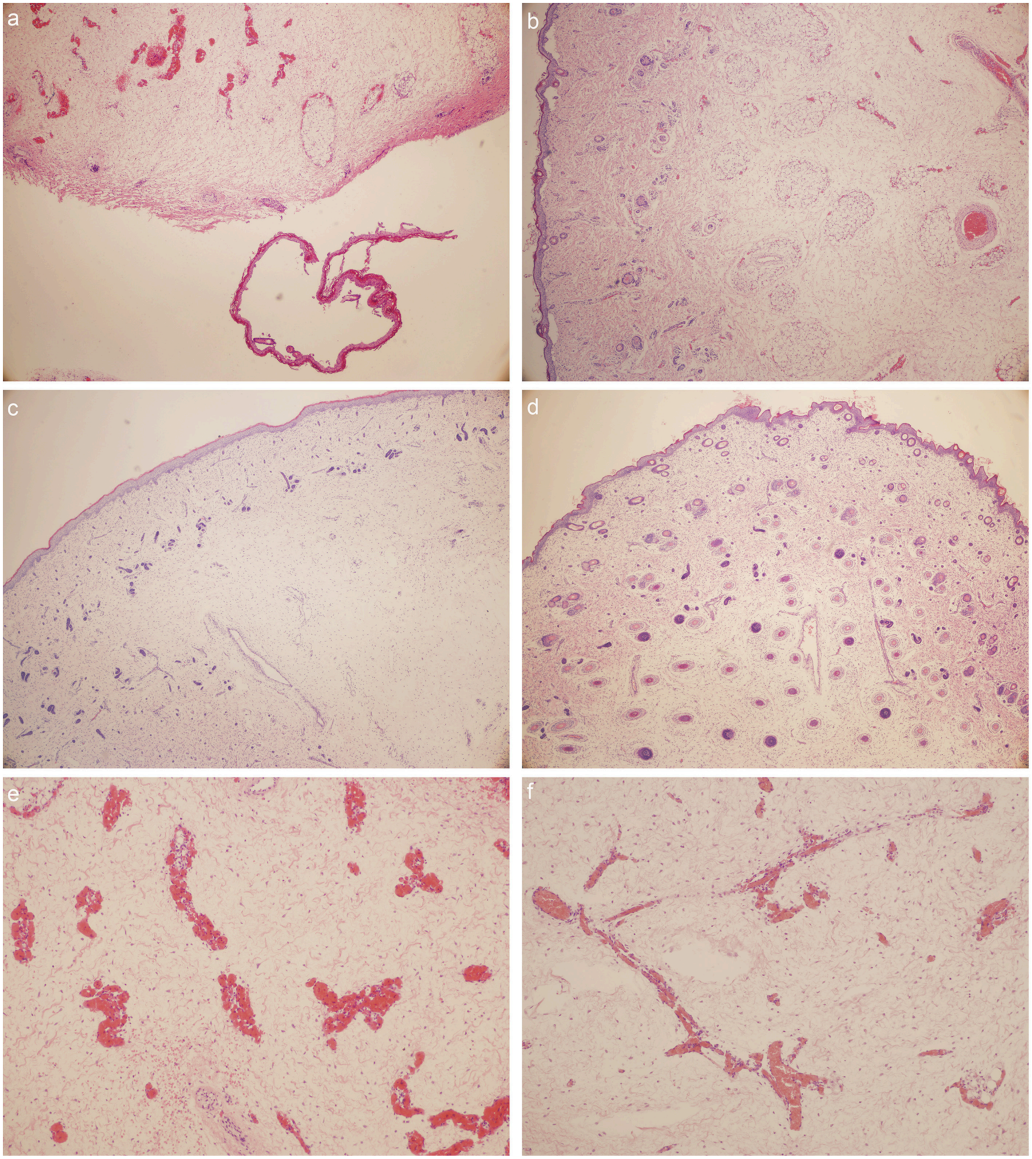
HE stain: **(a)** foot dorsum of MD child (40 times magnification); **(b)** lateral low-limb skin of MD child (40 times magnification); **(c)** foot dorsum of matched child (40 times magnification); **(d)** lateral low-limb skin of matched child (40 times magnification). **(e,f)** lymphatic vessels found within the field of vision **(e)** foot dorsum of MD child (100 times magnification); **(f)** lateral low-limb skin of MD child (100 times magnification).

**Venous abnormalities** (Figure 4)

1. Capillary hyperemia is severe in Milroy disease child.
2. Phlebectasia could be detected in MD child.
3. Vascular malformations could be detected in Milroy disease child.
4. The number of vascular endothelial cells and vascular smooth muscle cells decreased in MD child.

**Skin abnormalities** (Figure 4)

1. Large sheets of desquamated epidermis could be detected in MD child.
2. The numbers of cutaneous appendages reduced in MD child.

### Genotyping by Next-Generation Sequencing *(NGS)*, Sequence Analysis, and Cosegregation in the Family

Genomic DNA was isolated from peripheral blood samples from the family members using standard methods. NGS was applied to the MD child. Then, the other members in the family were verified by Sanger sequencing and cosegregation. The base pair numbers of mutation sites were determined according to the GenBank mRNA reference sequences.

**Figure 5:**
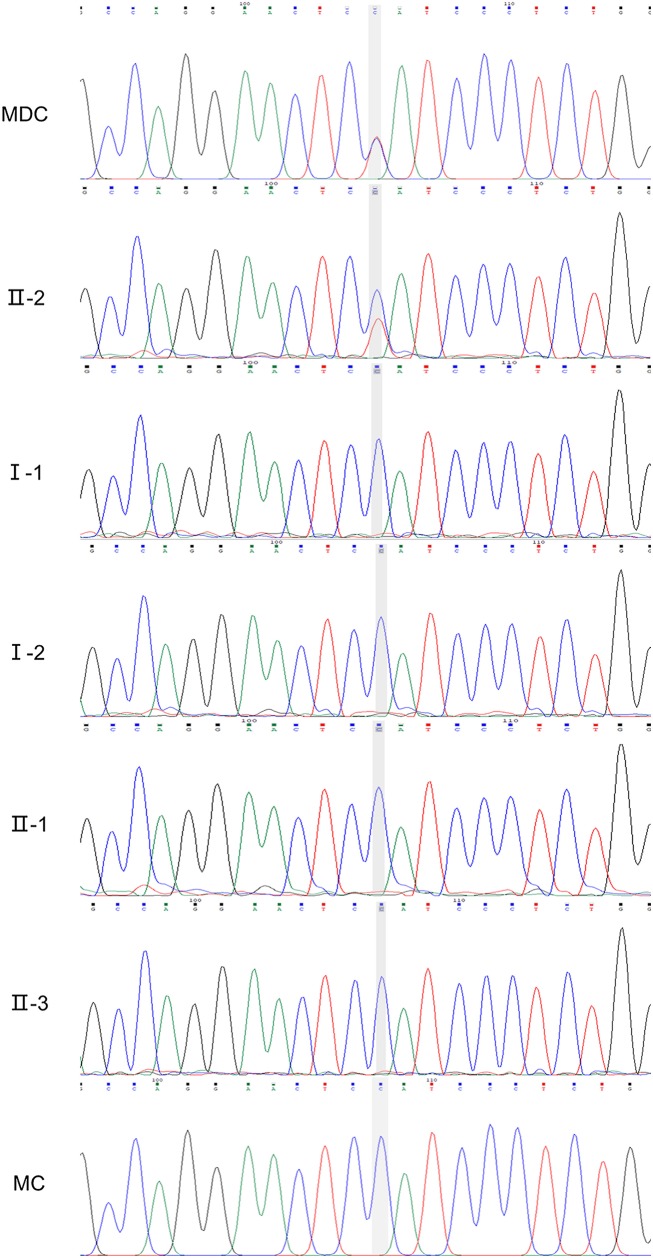
Sanger verification results of the family (II-2, FLT4: c.3075G>A). MDC, MD child; MC, matched child.

NGS revealed that the MD child has one heterozygous missense mutation in the FLT4: c.3075G>A *(p.M1025I)* in exon 22, a mutation which have not been described in Chinese ethnic. The result of the matched child showed normal in the FLT4 (Figure 5).

The mutation was subsequently confirmed by sanger sequencing of the family. The following cosegregation of the FLT4 alleles in the family pedigree confirmed that the c.3075G>A allele was paternal *(II-2)*.

## Discussion

MD is characterized by heredity, painless, slow progression of disease, and it is limited to lower limb edema. MD could be diagnosed according to the clinical characteristics and genetic analysis. The edema usually occurs from *(or before)* birth. In neonates the swelling tends to affect primarily the dorsum of the feet. The amount of edema varies from individuals. Other features associated with MD sometimes include: Hydrocele (37% of males); Prominent veins (23%); Upslanting toenails (14%);Papillomatosis (10%); Urethral abnormalities in males (4%) (Brice et al., 1993).

The mutation of VEGFR-3 *(FLT4)* is believed responsible for the lymphedema in MD. The protein is a member of the tyrosine kinase receptor family, which plays an important role in lymphangiogenesis. In MD, there is believed an abnormal accumulation of interstitial protein-rich fluid caused by congenital malformation of the lymphatic vessels (Tammela and Alitalo, 2010; Gezginc et al., 2012). Similar results have been confirmed through lymphangiography that there is local lymphangiogenesis insufficiency in the extremities of MD children (Rooke, 2003).

For human, the development of the lymphatic vascular system begins in the sixth to seventh week of embryonic life. Malformation of the lymphatic vessels could trigger an increase of the interstitial protein rich fluid, which subsequently results in insufficient lymphatic transport and drainage (Kitsiou-Tzeli et al., 2010). As a result, large amount of protein-rich fluid accumulates in tissue interstitial spaces, which makes skin, subcutaneous tissue, fibrous tissue hyperplasia, and oppression of lymphatics more difficult for lymphatic reflux, thus forming a vicious cycle. The skin thickened, hardened, getting rough and bulky, forming “elephant skin” swelling over time.

In our findings, the numbers of the lymphatic vessels in MD child decreased. The morphology and structure of lymphatic vessels was abnormal. These phenomena confirmed the evidences given by previous animal and cell studies of the FLT4 mutation (Rauniyar et al., 2018). But out of our expectation, the capillary and skin were also influenced in fact.

Capillary hyperemia and phlebectasia is severe in MD child. Vascular malformations could also be detected. And the number of vascular endothelial cells and vascular smooth muscle cells decreased in MD child. All the results indicated the mutation of FLT4 did not only affect the lymphatic formation, but also affect the blood vessels in human. Furthermore, large sheets of epidermis were desquamated. And the numbers of cutaneous appendages were also reduced. So concerning the connection between phenotype and genotype, we assume that mutation of FLT4 affect the angiogenesis and epidermis structure in human. While, this assumption still needs further evidence.

At present, radionuclide lymphatic imaging is the preferred method to observe the lymphatic system. Meanwhile, the b-ultrasonography, as a convenient and fast examination method, has still been widely used in the diagnosis and evaluation of lymphoedema in gravida (Matter et al., 2002). Unfortunately, there is no effective treatment for lymphedema until now. Conservative treatment methods include roasting therapy, intermittent compression therapy and so on. If the edema and fibrosis is aggravated, surgical treatment might be required (Becker et al., 2012). And the key to treat MD is figuring out how the lymphedema come into being, and we still need more evidences.

## Author Contributions

SZ and YW planned the study. SZ and XC conducted the surgery. SZ and LY conducted the histological staining. SZ and SW wrote the article. DM organized the photographs. SL and YW supervised the study.

### Conflict of Interest Statement

The authors declare that the research was conducted in the absence of any commercial or financial relationships that could be construed as a potential conflict of interest.

## Supplementary Material

Supplementary Video 1 The edema does not affect normal walking to the father patient.
